# Acupuncture for ICU patients: evidence, mechanisms, and implementation challenges

**DOI:** 10.3389/fneur.2025.1711600

**Published:** 2026-01-02

**Authors:** Pochen Li, Yang Wu, Yan Wu, Danxia Ge, Qianping Zhang, Yujiao Li, Fangyu Yu

**Affiliations:** 1 Intensive Care Unit, Ningbo Municipal Hospital of Traditional Chinese Medicine (TCM), Affiliated Hospital of Zhejiang Chinese Medical University, Ningbo, China; 2 Department of Respiratory, Ningbo Municipal Hospital of Traditional Chinese Medicine (TCM), Affiliated Hospital of Zhejiang Chinese Medical University, Ningbo, China

**Keywords:** acupuncture, analgesia and sedation, aseptic technique, delirium, gastrointestinal dysfunction, ICU-acquired weakness, intensive care unit, standardization of acupuncture

## Abstract

**Objective:**

To explore the clinical applications, mechanisms of action, and methodological and implementation challenges of acupuncture in intensive care unit (ICU) patients, to provide a theoretical foundation for its standardization and evidence-based advancement.

**Methods:**

A comprehensive analysis of recent clinical studies, mechanistic investigations, and consensus guidelines regarding acupuncture use among ICU patients was conducted. The review summarizes its efficacy in analgesia and sedation, ICU-acquired weakness (ICU-AW), delirium, and gastrointestinal dysfunction, while systematically addressing aspects of infection control, procedural standardization, and research design.

**Results:**

Multiple randomized controlled trials (RCTs) and systematic reviews indicate that acupuncture interventions can effectively reduce reliance on analgesic and sedative medications, shorten the duration of mechanical ventilation and ICU stay, reduce the incidence of ICU-AW and delirium, and improve gastrointestinal motility. Mechanistic studies suggest that acupuncture exerts its effects via several pathways, including activation of the vagus nerve–cholinergic anti-inflammatory reflex, modulation of central analgesic networks, restoration of hypothalamic–pituitary–adrenal (HPA) axis homeostasis, and enhancement of microcirculation. However, clinical implementation remains limited by infection-control concerns, procedural heterogeneity, and the overall low quality of evidence.

**Conclusion:**

Current evidence indicates that acupuncture is generally safe and exerts multisystem regulatory effects in ICU patients, supporting its potential as a complementary therapy in critical care. Nevertheless, its role should be further validated through rigorous multicenter trials, standardized operational frameworks, and strict aseptic protocols prior to its incorporation into routine ICU practice.

## 1 Introduction

With advances in extracorporeal life support technologies and precision monitoring, mortality in intensive care units (ICUs) has significantly declined (1). However, this survival benefit is often accompanied by complications such as persistent pain, delirium, ICU-acquired weakness (ICU-AW), and gastrointestinal dysmotility (2–5). These complications substantially impair functional recovery and quality of life. Recent multicenter data indicate that ICU-AW affects 40–50% of patients, creating a vicious cycle of muscle weakness, infection risk, and prolonged hospitalization (6). Conventional treatment strategies, dominated by opioids, benzodiazepines, muscle relaxants, and prokinetic agents, may alleviate symptoms but are frequently associated with adverse effects such as respiratory depression, drug resistance, and delirium (7). Consequently, there is an urgent need for non-pharmacologic interventions that have minimal side effects and are compatible with modern monitoring systems.

Acupuncture, characterized by its multi-target regulation, adaptability, and reproducibility, is gradually attracting the attention of critical care practitioners. Evidence from randomized controlled trials (RCTs), systematic reviews, and experimental studies suggests its potential benefits in analgesia and sedation, organ-function improvement, and rehabilitation, with a favorable safety profile (8–10). Nonetheless, limitations such as small sample sizes and the lack of standardized procedures persist. This review summarizes the clinical applications and mechanisms of acupuncture in ICU patients, with a particular focus on infection control and standardization of practice. It also outlines future research directions to provide a theoretical and practical framework for the interdisciplinary integration of acupuncture and critical care medicine.

In this review, the term “acupuncture” encompasses both manual needle stimulation and its contemporary electrical extensions, including electroacupuncture (EA)—which delivers low-frequency electrical currents through inserted needles—and transcutaneous electrical acupoint stimulation (TEAS), a non-invasive surface modality applying similar electrical parameters via adhesive electrodes. These techniques share common neurophysiological mechanisms but differ in invasiveness and quantifiability, aspects particularly relevant in the ICU setting.

## 2 Literature search strategy

This mini-review was informed by a targeted search of both English- and Chinese-language biomedical literature. We searched PubMed, Web of Science, Embase, and the Cochrane Library, as well as the major Chinese databases CNKI, WanFang Data, VIP, and SinoMed, from database inception to September 2025.

The search strategy combined controlled vocabulary (e.g., MeSH terms) and free-text terms related to acupuncture and critical care, including “acupuncture,” “electroacupuncture,” “transcutaneous electrical acupoint stimulation,” “TEAS,” “intensive care unit,” “critical care,” “mechanical ventilation,” “ICU-acquired weakness,” “delirium,” and “gastrointestinal dysfunction,” using Boolean operators (AND/OR).

We focused on randomized controlled trials, observational clinical studies, systematic reviews, and key mechanistic investigations relevant to the use of acupuncture in ICU and critically ill populations. Additional references were identified by manually screening the bibliographies of relevant articles and guidelines. Given the scope of this paper as a mini-review, a formal PRISMA-based systematic review process was not performed.

Nonetheless, basic characteristics of the included studies are summarized in the Supplementary material.

## 3 Clinical application of acupuncture in ICU patients

### 3.1 Analgesia, sedation, and weaning from mechanical ventilation

Analgesia and sedation represent a double-edged sword in managing mechanically ventilated critically ill patients. While necessary to prevent unplanned extubation and maintain hemodynamic stability, excessive use of sedatives and analgesics can delay weaning, induce delirium, and contribute to ICU-AW (11, 12). Early extubation strategies emphasize the principle of “less is more” in drug administration (13). However, under conditions of cytokine storm, hypermetabolism, and multiple organ support, the use of opioids and benzodiazepines is often intensified, which in turn increases the risk of respiratory depression, tolerance, and delirium (11).

With growing clinical experience and supporting evidence, acupuncture has been incorporated into enhanced recovery after surgery protocols in ICU settings across several countries and regions. Accumulating evidence supports its potential role in analgesia, sedative sparing, and ventilator management (14, 15).

RCTs have demonstrated that combining EA or TEAS with standardized analgesia and sedation protocols significantly reduces the daily doses of propofol and fentanyl (16, 17). A 2024 systematic review and meta-analysis comprising 10 RCTs involving 756 critically ill patients further supported these findings. Compared with conventional or sham acupuncture controls, acupuncture interventions shortened the duration of mechanical ventilation by an average of 2.06 days (MD = −2.06, 95% CI −3.33 to −0.79, *p* = 0.001), reduced ICU length of stay by 1.26 days (MD = −1.26, 95% CI −2.00 to −0.53, *p* = 0.0008), and decreased the 28-day all-cause mortality risk by 33% (RR = 0.67, 95% CI 0.47–0.94). Subgroup analyses indicated that EA and TEAS showed the greatest efficacy, suggesting that continuous and quantifiable electrical stimulation may be a key factor contributing to their synergistic analgesic and sedative effects (18).

### 3.2 ICU-AW and early rehabilitation

ICU-AW is a multifactorial complication arising from prolonged bed rest, systemic inflammation, neuromuscular blockade, and sedative use. It typically presents as symmetric proximal muscle weakness in the limbs, significantly impairing voluntary movement and long-term functional recovery. Previous studies have reported an incidence of 43–50% ICU-AW among critically ill patients, with a markedly higher risk in those requiring mechanical ventilation for more than 7 days. Affected patients often experience a prolonged hospital stay of approximately 5–8 days and have a significantly higher 90-day readmission rate (19). Based on these findings, the Society of Critical Care Medicine (SCCM) and international early rehabilitation consensus guidelines recommend incorporating maintenance of muscle strength and functional training into early ICU intervention protocols (20, 21).

In recent years, acupuncture in China has become an integral component of comprehensive rehabilitation strategies for the prevention and treatment of ICU-AW. Several small-scale clinical trials have demonstrated its potential to enhance muscle strength recovery and improve neuromuscular conduction. In a randomized controlled trial conducted by Yu et al., 80 patients with ICU-AW were assigned to either a standard rehabilitation group or a rehabilitation plus acupuncture group. The intervention group received acupuncture at Zusanli (ST36), Quchi (LI11), Hegu (LI4), Yinlingquan (SP9), and Fenglong (ST40) every other day over a one-month period, with each session lasting 30 min. Results showed that patients in the acupuncture group achieved significantly higher Medical Research Council (MRC) muscle strength scores than those in the control group (*p* < 0.01), and their average duration of mechanical ventilation was shorter by approximately 2.17 days (22).

Similarly, Liu et al. reported that acupuncture combined with early bedside exercise training increased muscle cross-sectional area (CSA) and quadriceps thickness in ICU patients, which was associated with higher MRC scores. These findings suggest that acupuncture exerts beneficial effects on both structural and functional aspects of muscle recovery. Additionally, patients in the acupuncture group experienced significantly shorter ICU stays and mechanical ventilation durations than those in the control group (23).

Notably, some supporting evidence is derived from dissertations with limited external validation, and should be interpreted as preliminary findings pending confirmation from larger multicenter trials.

### 3.3 Mitigation of delirium and cognitive impairment

ICU delirium is an acute disturbance in cerebral function whose incidence ranges from 45 to 80% in mechanically ventilated patients and is closely associated with increased one-year mortality, persistent cognitive deficits, and rising healthcare costs (24). Conventional pharmacologic approaches—including titrated dexmedetomidine, propofol, and antipsychotics—neither markedly reduce delirium incidence nor avoid adverse effects such as bradycardia, hypotension, and delayed extubation (25). Consequently, the 2022 KSCCM update of international critical-care guidelines recommends prioritizing multimodal, non-pharmacologic preventive strategies (26).

Emerging clinical and translational studies suggest that acupuncture and its derivatives can modulate neurotransmitter release, suppress neuroinflammation, and restore circadian rhythm homeostasis. These multitarget effects make acupuncture a promising adjunct for delirium prevention and treatment. In a double-blind, RCT conducted in 2025, 24 ICU patients with Intensive Care Delirium Screening Checklist (ICDSC) scores ≥4 were enrolled to assess the efficacy of press-needle patch retention therapy. Patients in the intervention group received press needles, while the control group received placebo patches. The results showed that during the first week of treatment, the median number of delirium-free days was significantly higher in the intervention group than in the control group [median (IQR): 5 (4–5) vs. 2 (0–4); *p* = 0.025]. This improvement in delirium status persisted throughout the 4-week follow-up period (27).

A multicenter RCT conducted in China enrolled 210 high-risk patients transferred to the ICU after major abdominal surgery to receive TEAS combined with auricular seed pressure or standard care. The incidence of delirium on the day of surgery was 5.7% (6/105) in the intervention group and 15.2% (16/105) in the control group (RR = 0.38; 95% CI 0.15–0.92; *p* = 0.024) (28). Meta-analysis further confirmed that TEAS significantly reduces both the incidence of postoperative delirium (RR = 0.40, 95% CI 0.29–0.55, *p* < 0.00001), and shortens the duration of delirium (MD = −0.97; 95% CI −1.72 to −0.22; *p* = 0.01) (29).

### 3.4 Improvement of gastrointestinal function

Gastrointestinal dysfunction ranks among the most common complications in ICU patients and exhibits marked heterogeneity. Clinical manifestations range from hypomotility conditions—such as gastric retention, functional constipation, and absent bowel sounds—to hypermotility states including stress-related diarrhea and increased intestinal permeability. Studies have shown that constipation affects more than 70% of critically ill patients, while diarrhea occurs in at least 40% (30). Both conditions often alternate in the same individual and are driven by dysbiosis, medication use, nutritional support strategies, and underlying disease. Conventional therapy relies heavily on prokinetic agents, laxatives, or antidiarrheals; however, these unidirectional approaches fail to address the dynamic pathophysiology and may cause dependence, intestinal spasm, or electrolyte disturbances. As a traditional Chinese intervention that “harmonizes yin-yang and the zang-fu organs,” acupuncture has demonstrated bidirectional regulation of gut motility in modern clinical research and has become an important adjunct in managing gastrointestinal function in the critically ill.

Clinical evidence supporting acupuncture for the treatment of functional constipation has been steadily accumulating. An RCT conducted in 2020 enrolled 98 ICU patients with constipation, receiving daily acupuncture at Zusanli (ST36), Tianshu (ST25), and Shangjuxu (ST37) for six consecutive days. Results showed that the defecation rate on day 6 was significantly higher in the acupuncture group than in the conventional care group (91.84% vs. 71.34%, *p* < 0.05). In addition, acupuncture demonstrated notable effectiveness in alleviating opioid-induced gastric hypomotility. A meta-analysis evaluating acupuncture for opioid-induced constipation reported an overall symptom relief rate of 86.8% in the acupuncture group, which was significantly higher than that of the control group (78.9%; RR = 1.10, 95% CI 1.03–1.18). The study also highlighted that improvement in constipation significantly enhanced patients’ quality of life (31).

Acupuncture has also demonstrated distinct advantages in the management of diarrhea. In a prospective observational study conducted in 2024 by Fujian University of Traditional Chinese Medicine, ICU patients with enteral nutrition–associated diarrhea were treated with EA at Guanyuan (CV4), Zusanli (ST36), and Zhongwan (CV12). Results showed that the seven-day incidence of diarrhea in the intervention group was significantly lower than that in the control group (*p* < 0.05). Notably, no acupuncture-related adverse events were reported throughout the treatment period (32).

**Figure 1:**
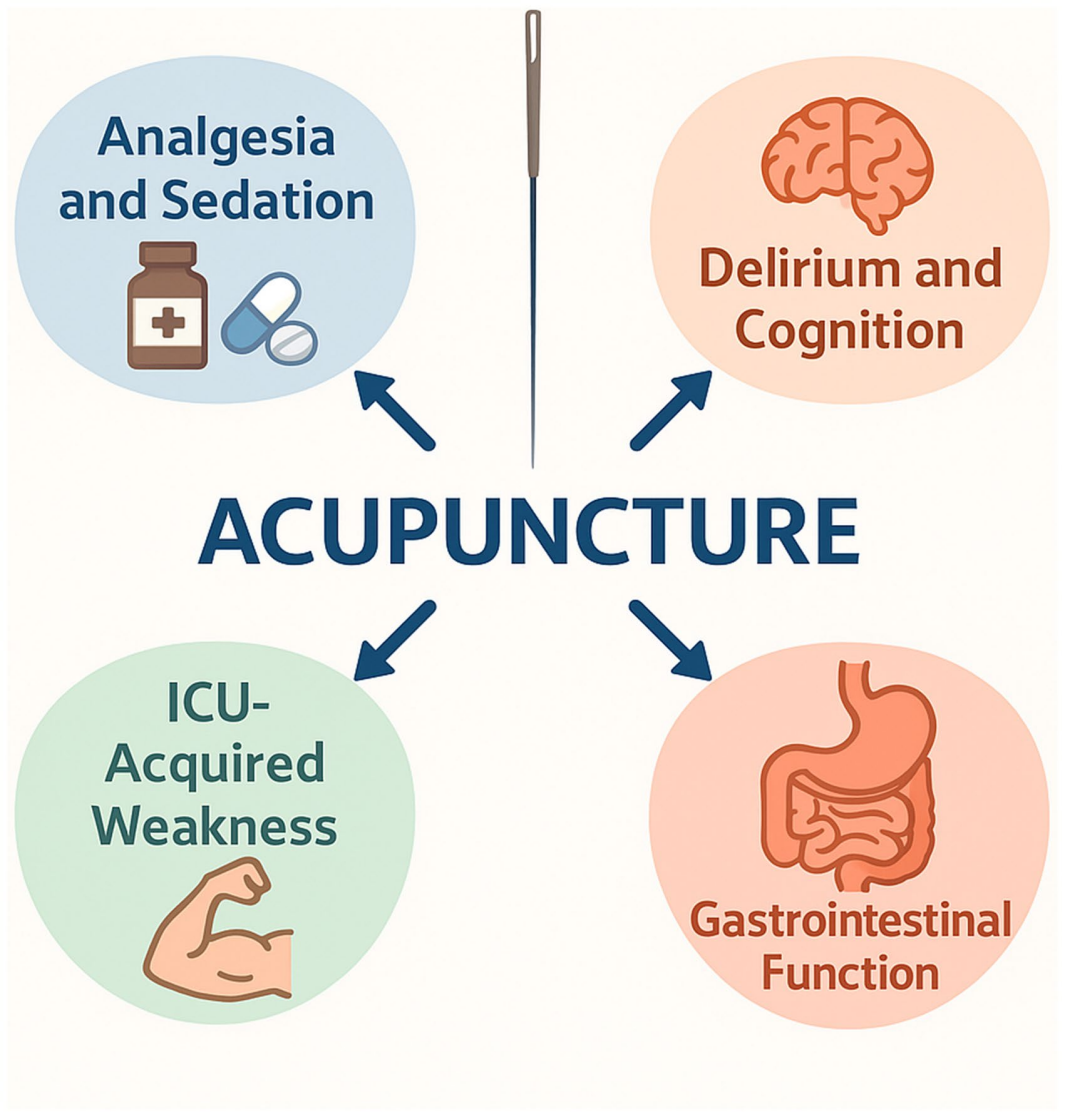
Clinical applications of acupuncture in ICU patients. Acupuncture has been applied in the intensive care unit (ICU) as an adjunctive intervention with multiple clinical benefits. Major applications include: (1) analgesia and sedation, helping reduce sedative and opioid consumption; (2) prevention and rehabilitation of ICU-acquired weakness (ICU-AW); (3) mitigation of delirium and improvement of cognitive outcomes; and (4) regulation of gastrointestinal function, such as alleviation of constipation or diarrhea.

Overall, acupuncture exhibits broad-spectrum efficacy in modulating gastrointestinal function in ICU patients. It is suitable not only for hypomotility conditions such as functional constipation and gastric retention, but also for hypermotility states such as leakage-type or enteral nutrition–associated diarrhea. Furthermore, it offers key advantages including high safety, bedside feasibility, and independence from pharmacologic agents (Figure 1).

## 4 Mechanisms of acupuncture intervention in ICU patients

**Figure 2:**
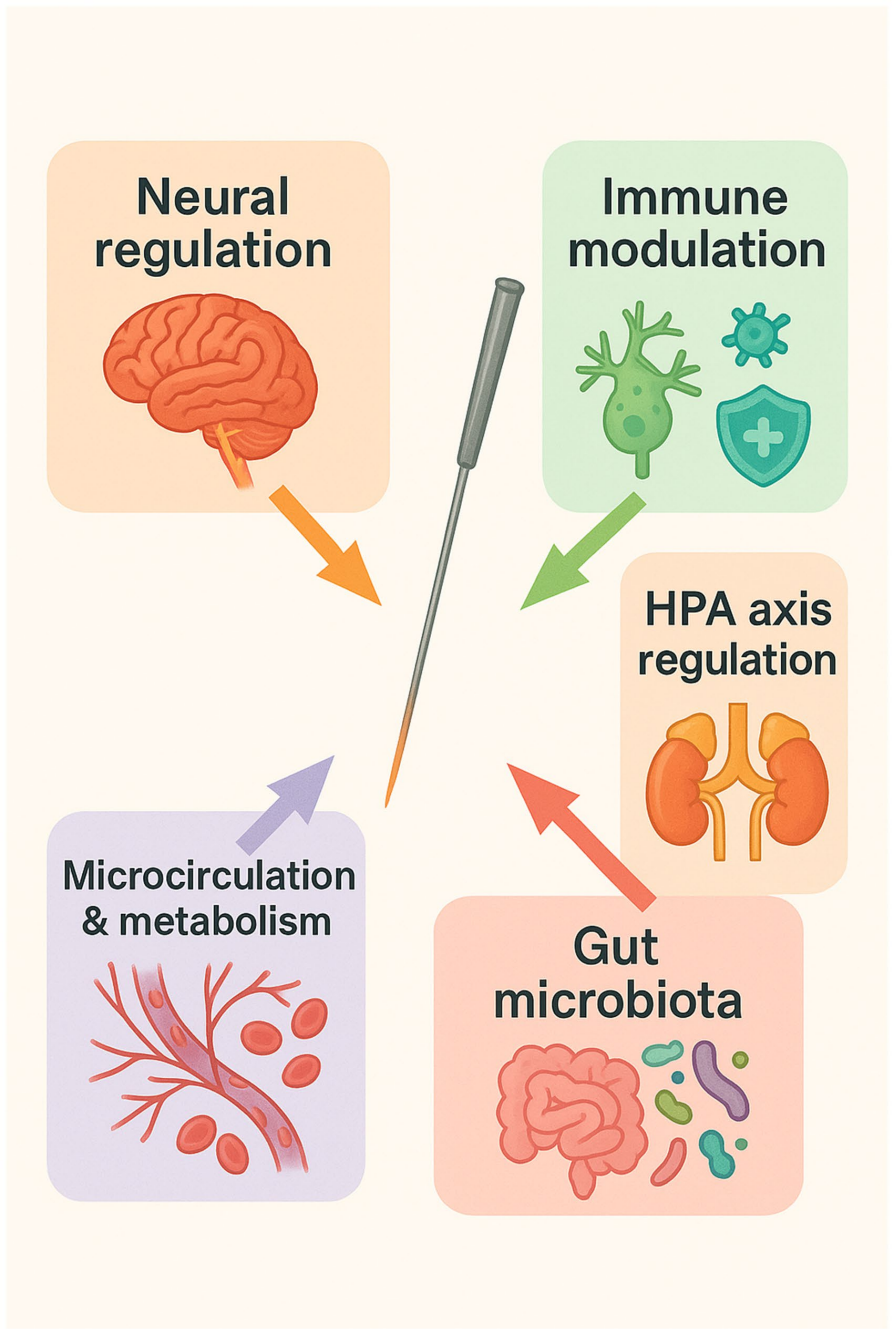
Mechanisms of acupuncture intervention in ICU patients. Acupuncture exerts therapeutic effects in critical illness through multiple systemic pathways: (1) neural regulation via modulation of central and peripheral nervous activity; (2) immune modulation, particularly through the cholinergic anti-inflammatory pathway; (3) endocrine balance, restoring hypothalamic–pituitary–adrenal (HPA) axis feedback; (4) improvement of microcirculation and muscle metabolism; and (5) regulation of gut microbiota.

The role of acupuncture in ICU patients extends well beyond symptomatic relief, functioning instead within a complex, multidimensional regulatory network. Growing evidence from basic and translational studies suggests that acupuncture modulates neural, immune, and endocrine systems in a coordinated manner to exert its therapeutic effects. By attenuating inflammation, maintaining physiological homeostasis, and promoting tissue repair, acupuncture establishes a strong biological rationale for its integration into critical care practice (Figure 2).

First, acupuncture suppresses systemic inflammatory responses by activating the vagus nerve-cholinergic anti-inflammatory pathway (CAP). Animal studies have confirmed that EA at classical immune-related points significantly enhances the expression of α7-nicotinic acetylcholine receptors (α7nAChRs) on splenic macrophages (33). Clinical evidence also indicates that continuous EA can significantly lower inflammatory markers in ICU patients—such as C-reactive protein (CRP) and procalcitonin (PCT)—suggesting an anti-inflammatory regulatory role (34).

Furthermore, acupuncture modulates pain pathways via the peripheral–spinal–central nervous system (CNS) axis, contributing to synergistic sedation and analgesia. Stimulation of Aδ and C fibers by acupuncture transmits signals through the spinothalamic tract to the central nervous system. This subsequently triggers the release of endogenous opioids such as *β*-endorphin and enkephalin, thereby elevating pain thresholds and attenuating stress responses (35). Additionally, acupuncture enhances the levels of neurotransmitters such as serotonin (5-HT) and norepinephrine (NE), reinforcing central analgesia and emotional stability (36). These mechanisms help explain clinical observations that acupuncture reduces the required doses of sedatives like midazolam and fentanyl in ICU settings and mitigates drug-related adverse effects.

With respect to neuroendocrine homeostasis, acupuncture exerts regulatory effects on the hypothalamic–pituitary–adrenal (HPA) axis. Critical illness often leads to HPA overactivation, resulting in a state of sustained hypercortisolemia that suppresses immunity, promotes muscle protein catabolism, and damages neurons (37). Studies have shown that acupuncture can inhibit excessive secretion of adrenocorticotropic hormone (ACTH) and cortisol (CORT), restoring negative feedback balance in the HPA axis, thereby alleviating stress overload and improving neurobehavioral conditions such as delirium and sleep disturbances (38).

Acupuncture further enhances microcirculation and tissue metabolism. In wound healing and in interventions targeting ICU-AW, EA enhances nitric oxide (NO) production, dilates capillary networks, improves local perfusion, and accelerates granulation tissue formation and nutrient delivery (39). Animal studies reveal that acupuncture upregulates mitochondrial energy pathways (e.g., PGC-1α, AMPK) and downregulates oxidative stress markers, contributing to tissue repair and antioxidant defense. These findings highlight acupuncture’s regulatory potential in both local and systemic metabolic environments in ICU patients (40).

Additionally, accumulating evidence indicates that acupuncture modulates systemic inflammation and neuroimmune interactions via the gut–brain axis. EA at Zusanli (ST36) and Tianshu (ST25) upregulates intestinal tight junction proteins such as ZO-1 and occludin and concurrently suppresses pro-inflammatory cytokines such as tumor necrosis factor-alpha (TNF-*α*) and interleukin-6 (IL-6), thus preserving mucosal barrier integrity and preventing bacterial translocation (41, 42). Additionally, acupuncture can modulate gut microbiota homeostasis. Although large-scale clinical trials are still lacking, animal studies have shown that acupuncture counteracts antibiotic-induced dysbiosis, modulates the Firmicutes/Bacteroidetes ratio, and restores microbial diversity (43). Furthermore, acupuncture reshapes gut microbial composition by increasing the relative abundance of short-chain fatty acid (SCFA)-producing bacteria such as Bifidobacterium and Lactobacillus while reducing potentially pathogenic taxa. SCFAs act as metabolic signaling molecules that regulate microglial activation and modulate HPA axis activity, forming a key biochemical link between the gut and the brain (44, 45).

In summary, the mechanisms of acupuncture in ICU patients encompass neural regulation, immune modulation, endocrine balance, tissue metabolism, and microcirculatory support, thereby demonstrating substantial integrative potential. These multi-pathway and synergistic effects are well aligned with the complex, multi-system pathology of critical illness, establishing a robust biological basis for acupuncture as a sustainable, non-pharmacologic intervention in the ICU. Future studies should further elucidate the hierarchical relationships and key nodes among these mechanisms to facilitate the development and clinical implementation of precision acupuncture protocols.

## 5 Key clinical issues and future directions

### 5.1 Infection prevention and aseptic workflow

ICU patients are characterized by immunosuppression, increased infection risk, and impaired skin–mucosal integrity; thus, even minor lapses during any invasive procedure can precipitate life-threatening sepsis (46). Therefore, strict adherence to aseptic principles is the foremost prerequisite for the safe integration of acupuncture into critical care practice.

Each patient should receive acupuncture with a dedicated, single-use needle that complies with ISO-certified sterile standards. If the packaging is opened and the procedure is delayed for more than 30 min, the needle must be discarded and replaced to ensure safety. Operators must perform hand hygiene before and after each procedure. The insertion site should be thoroughly disinfected using either 2% chlorhexidine or 75% ethanol, applied for a minimum of 30 s and allowed to dry completely (47). Following needle removal, firm pressure should be applied for 1 to 3 min depending on the patient’s coagulation status, and the area should then be covered with a semi-permeable dressing for 24 h. Each clinical site must maintain an “Acupuncture Quality and Safety Log” to document daily observations of puncture-site erythema, exudation, or other abnormalities. Any suspected acupuncture-related infection must prompt immediate root-cause investigation and microbiological culture testing within 24 h.

Only through a management model of standardized operation and continuous surveillance can the risk of acupuncture-related infection in the ICU be minimized and a reproducible operational baseline established for multicenter evidence accumulation.

### 5.2 Standardization of procedures and the dose–response relationship

Analyses from multicenter studies have demonstrated considerable variability in the reported efficacy of acupuncture, largely attributable to the absence of a quantifiable and standardized “dosing” framework. To mitigate this procedural heterogeneity, future clinical trials could place greater emphasis on the systematic documentation of objective outcome measures—such as BIS (Bispectral Index), RASS (Richmond Agitation–Sedation Scale), and MRC scores—while concurrently recording detailed procedural information, including acupoint selection, needle insertion depth, electrical stimulation parameters, session duration, and treatment frequency.

To enhance reproducibility across studies, it may be worthwhile to develop a more quantitative “dose–response” model to characterize acupuncture exposure. Potentially relevant parameters could include: (1) electrical frequency and waveform (e.g., alternating 2–10 Hz low-frequency versus continuous 100 Hz high-frequency stimulation); (2) current intensity adjusted to produce a mild, visible muscle twitch or limited to the patient’s tolerance; (3) needle retention time, typically between 20 and 40 min per session; and (4) cumulative treatment frequency, which may vary from daily to every other day depending on clinical context.

Defining approximate parameter ranges might help improve inter-study comparability and facilitate future meta-analytic integration. In parallel, establishing a multicenter consensus registry that collects and harmonizes stimulation parameters alongside standardized clinical outcomes (e.g., BIS, RASS, and MRC scores) could represent a pragmatic first step toward identifying reproducible “dose metrics” for acupuncture within ICU settings.

### 5.3 Study design and evidence-based evaluation

Future clinical research on acupuncture in the ICU should be grounded in a rigorous framework encompassing multicenter, randomized, blinded, and controlled trials. At the protocol development stage, precise estimations of sample size, attrition rate, and effect size should be carefully estimated.

The intervention group should receive a standardized acupuncture prescription, while control groups may employ sham acupuncture, electrode patch placebo, or best standard care. Primary early-stage outcomes may include ventilator days, 48-h reduction rate of sedative dosage, and delirium incidence density. Long-term outcomes should encompass 90-day all-cause mortality, Barthel Index scores, ICU-AW incidence, and total medical costs. To enhance external validity, prospective registry cohorts and electronic health record (EHR)-based data linkage should be established for real-world Supplementary material.

Reporting should adhere strictly to the CONSORT, STRICTA, and SPIRIT-AI guidelines. Evidence grading should follow the GRADE framework, with sensitivity analyses to assess heterogeneity, publication bias, and indirectness. An independent Data and Safety Monitoring Board (DSMB) should be established to oversee interim analyses and ensure ethical compliance throughout the study.

### 5.4 Research limitations and challenges

Despite notable progress in both clinical and basic research on acupuncture in critical care settings over recent years, the current body of evidence continues to face several key methodological and practical limitations:

First, most available studies are single-center, small-sample, or pilot trials with limited geographic, ethnic, and disease-type representation, undermining external validity. In addition, heterogeneous sham-acupuncture methods and the practical difficulty of blinding elevate the risk of performance bias.

Second, there is no unified guidance on acupoint localization, needling depth, or electrical-stimulation settings. Even a single nominal point—such as Zusanli (ST36)—can vary significantly in muscle layer targeting, needling angle, and retention time, thereby weakening inter-study comparability and obscuring dose–response analyses.

Third, most existing acupuncture-related ICU studies have primarily focused on short-term clinical endpoints, while long-term outcomes such as 90-day or 1-year survival, functional recovery, and neurocognitive performance remain insufficiently assessed. Future investigations should therefore include extended follow-up evaluations to comprehensively determine the durability and clinical relevance of acupuncture’s therapeutic effects in critically ill patients.

Fourth, mechanistic investigations have concentrated mainly on inflammatory modulation and endogenous opioid release, with scant exploration of broader systems-level pathways such as metabolic reprogramming, neural-network plasticity, and the gut–brain axis.

Fifth, cross-national legal, ethical, and cultural differences hinder global implementation. Some countries lack explicit regulations for administering acupuncture to deeply sedated patients, and standardized templates for proxy informed consent remain undeveloped.

## 6 Conclusion

Acupuncture, as a non-pharmacologic intervention with broad regulatory potential, is gaining growing recognition and clinical support in intensive care. Current evidence shows that it can safely and effectively reduce dependence on analgesic and sedative drugs, facilitate ventilator weaning, mitigate ICU-AW, decrease the incidence of delirium, and improve gastrointestinal function. These benefits position acupuncture as a reproducible, low-risk, and potentially individualized adjunct, particularly valuable when conventional therapies are limited by adverse effects. Mechanistically, acupuncture exerts system-level effects through multi-target, multi-pathway actions—such as activating the vagus nerve–cholinergic anti-inflammatory pathway, modulating central analgesic networks, re-balancing HPA axis feedback, and enhancing microcirculation—suggesting a plausible biological rationale.

Despite these advantages, significant challenges remain, particularly infection control. ICU patients often exhibit immunosuppression, compromised skin integrity, and the presence of multiple catheters, thereby necessitating rigorous adherence to aseptic protocols. Embedding acupuncture within a closed-loop framework—that is, standardized procedures combined with continuous infection surveillance—is critical to ensure both safety and efficacy.

Future research should prioritize large multicenter RCTs, establish standardized operating procedures and dose–response frameworks, and incorporate real-world data with long-term outcome measures. Parallel efforts should deepen mechanistic exploration through multi-omics approaches and address ethical and regulatory considerations. Such advances will be pivotal in transitioning acupuncture from an adjunctive therapy to an integral component of ICU care, fostering a safe, evidence-based, and standardized paradigm for integrative critical care.
