# Supplementary material for: Acupuncture for ICU patients: evidence, mechanisms, and implementation challenges
Source: Front Neurol. 2026 Jan 2;16:1711600. doi: 10.3389/fneur.2025.1711600 (PMC12807917; doi:10.3389/fneur.2025.1711600)
Supplement: Supplementary file 1 [file Table_1.docx]

Basic characteristics of the included studies

| First author (year) | Section | Study model | n | Study design | Acupuncture modality | Main conclusion |
| --- | --- | --- | --- | --- | --- | --- |
| Chen J 2023 | Section 2.1 | Clinical | 80 | RCT | Traditional acupuncture | Acupuncture treatment can improve breathing ability of patients with respirator dependence in respiratory care center. |
| Nayak 2008 | Section 2.1 | Clinical | 12 | A pilot study | Electroacupuncture (EA) | Study showed significant reduction in the dose of propofol required for sedation in critically ill patients. |
| Wang T 2018 | Section 2.1 | Clinical | 60 | RCT | Transcutaneous electrical acupoint stimulation (TEAS) | TEAS stimulation exhibits time-dependent effects; administering it 30 minutes prior to surgery reduces the intraoperative requirement for remifentanil but has no significant impact on the dosage of other medications. |
| Eyal Ben-Arie 2024 | Section 2.1 | Clinical | 756 | Systematic review and meta-analysis | Traditional acupuncture | This study identified a noteworthy reduction in the total Mechanical Ventilation days and time spent in the ICU. |
| Yu J 2019 | Section 2.2 | Clinical | 80 | RCT | Traditional acupuncture | Acupuncture combined with Chinese herbal medicine significantly enhances limb muscle strength in ICU-AW patients. |
| Liu Z 2018 | Section 2.2 | Clinical | 44 | RCT | EA | EA stimulation improves muscle strength and oxygenation levels in mechanically ventilated ICU-AW patients, and can slow the rate and extent of muscle atrophy. |
| Kao P 2025 | Section 2.3 | Clinical | 24 | RCT | Press tack needles | This study suggests that press tack needle acupuncture may be an effective adjunctive intervention for reducing ICU delirium. |
| Fan Q 2022 | Section 2.3 | Clinical | 210 | RCT | EA+auricular acupressure | Reduced the incidence of postoperative in-hospital delirium compared with standard care. |
| Huang K 2023 | Section 2.3 | Clinical | 991 | Systematic review and meta-analysis | TEAS | TEAS could reduce the incidence of postoperative delirium and shorten the duration of postoperative delirium. |
| Han C 2021 | Section 2.4 | Clinical | 656 | Systematic review and meta-analysis | Traditional acupuncture | The co-intervention of acupuncture with drugs improves the outcomes ofopioid-induced constipation patients better than a single strategy. |
| LI Y 2022 | Section 2.4 | Clinical | 60 | RCT | Traditional acupuncture | Acupuncture treatment significantly improves ICU patients' tolerance to enteral nutrition, reduces the incidence of enteral nutrition-associated diarrhea, and improves stool consistency. |
| Huang C 2012 | Section 3 | Animal | - | Controlled animal experiment | EA | 1. The interaction between β-endorphin and cytokines may mediate the regulation of immune function by electroacupuncture. 2. α7 nAChR may participate in the regulation of immune function by 100Hz electroacupuncture. |
| Xian J 2023 | Section 3 | Clinical | 1099 | Systematic review and meta-analysis | Traditional acupuncture | Acupuncture treatment can reduce levels of multiple inflammatory factors. |
| Lan Y 2024 | Section 3 | Animal | - | Controlled animal experiment | EA | EA activates AMPK through CB2R, enhancing β-END expression in inflamed skin to alleviate inflammatory pain. |
| Zheng J 2024 | Section 3 | Animal | - | Controlled animal experiment | EA | EA alleviates HPA axis hyperactivity and anxiety-like behaviors caused by surgical trauma through inhibition of Nesfatin-1/ERK/CREB pathway in the hypothalamus. |
| Guo K 2024 | Section 3 | Animal | - | Controlled animal experiment | Traditional acupuncture | Acupuncture intervention promotes brain energy metabolism and mitochondrial biogenesis while slowing neuronal apoptosis. This mechanism involves activation of the AMPK/PGC-1α axis, with AMPK serving as a key therapeutic target. |
| Wang L 2020 | Section 3 | Animal | - | Controlled animal experiment | EA | EA can relieve chronic experimental colitis, and this effect may depend on activation of the MAPK signaling pathway through modulation of the gut microbiota to preserve the intestinal barrier. |
| Lv Z 2022 | Section 3 | Animal | - | Controlled animal experiment | Traditional acupuncture | Serum metabolomics analysis revealed that acupuncture treatment modulates differential metabolites including N-methylnicotinamide, beta-glycerophosphoric acid, geranyl acetoacetate, serotonin, and the biosynthetic pathways of phenylalanine, tyrosine, and tryptophan. It also affects taurine, hypotaurine, and beta-alanine metabolic pathways. |
